# Informal care relationships and residential aged care recommendations: evidence from administrative data

**DOI:** 10.1186/s12877-017-0656-4

**Published:** 2017-12-19

**Authors:** Jeromey B. Temple, Marijan Jukic, Briony Dow

**Affiliations:** Demography and Ageing Unit, Melbourne School of Population and Global Health, University of Melbourne, 207 Bouverie St, Melbourne, VIC 3010 Australia

**Keywords:** Informal care, Residential facilities, Gender, Aged care assessments

## Abstract

**Background:**

The Australian government recognises the importance of informal care to enable ageing in place. Yet, few multivariable studies have examined aspects of informal care that alter the probability of entry to residential care in Australia. Existing Australian and international studies show differing effects of informal care on entry to residential care.

**Methods:**

We utilise unique administrative data on aged care assessments collected from 2010 to 2013, consisting of 280,000 persons aged 65 and over. Logistic regression models were fitted to measure the propensity to be recommended care in a residential care setting, disaggregated by characteristics of informal care provision.

**Results:**

Providing some explanation for the divergent findings in the literature, we show that close familial carer relationships (partner or child) and coresidence are associated with recommendations to live in the community. Weaker non-coresidential friend or neighbour carer relationships are associated with recommendations to live in residential care for women, as are non-coresidential other relatives (not a child, partner or in-law) for both males and females. Non-coresident carers who are in-laws (for females) or parents have no impact on assessor recommendations. Despite these significant differences, health conditions and assistance needs play a strong role in assessor recommendations about entry to residential care.

**Conclusion:**

Co-resident care clearly plays an important protective role in residential care admission. Government policy should consider the need for differential supports for co-resident carers as part of future aged care reform.

## Background

Since the early 1980’s, the Australian Government’s aged care policy has consistently sought to assist older people to remain in their own homes (private dwellings) in preference to residential care. Fortunately, this is consistent with the preferences of older people [1]. The government has also long recognised the importance of informal carers in supporting older people at home, with family caregivers being considered a client of the home and community care service since its inception in 1986 and their needs assessed accordingly.

Indeed, informal caregiving is very prevalent in Australia. Approximately 2.7 million people (out of a population of almost 24 million), provided informal care in Australia, with two thirds of carers being female [2, 3]. In 2015, the economic value of the contribution of informal carers was valued at about $60 billion annually [4]. For older people, 80% of older Australians receiving assistance in the community receive care from informal caregivers [5].

Despite a recognition of the importance of informal care to the Australian government and older Australians themselves, there have been few multivariable studies examining the role of informal care as a risk factor to residential care entry. Although some studies have investigated proxy measures of informal care – such as living arrangements, marital status and family structure - these are imprecise measures of actual informal care received by older persons. Some Australian studies have shown that indicators of informal care, such as having living children and living arrangements were not significant predictors of entry to residential care [6]. However, marital status was significant for entry to care, and effects differed by gender. Other studies have also found that living alone was not significant for aged care placement, however this sample was geographically limited [7].

Within the international literature, studies have also provided mixed findings – due to differences in measures, as well as policy and country differences. Poor social support of older persons (an important proxy of care) has been found to be associated with entry to residential care in US studies [8, 9]. Other US studies have found that for disabled older persons, receipt of assistance from a child with basic personal care reduced the risk of nursing home entry [10]. In contrast, some US studies have found that receiving informal care, increases the risk of institutionalization [9]. UK studies have shown that persons who lived in a family with a spouse or a never married child had significantly lower risk of institutionalization than those living alone and that marital status is more important for men than for women [11].

In their key piece of analysis, Gaugler and colleagues argue that the associations between family assistance, informal care and residential care institutionalisation are complex and require detailed measures on the type, source and quality of informal care [12]. Utilising unique administrative data and detailed measures of informal caregiving, we seek to examine:

- After accounting for known predictors of residential care entry, does informal care change the likelihood of aged care assessors’ recommendations to residential care, and
- How does the older person’s family and social relationship and co-residence with the carer alter recommendations to residential care?

### Background on the Australian system

In Australia, eligibility for government funded home or residential care is governed by the Aged Care Assessment Program (ACAP). The objective of the ACAP program is to assess care needs and assist frail clients to access appropriate care. Through ACAP, Aged Care Assessment Teams (ACAT) make recommendations about individuals’ care setting, including, transitioning into residential care from the general community. The recommended long term care setting can include living in the community (eg. private residence, independent living) with home care package support, in residential care, hospitals or other types of institutional care. The ACAP assessment is a compulsory, formal step in obtaining access to government subsidised home and residential care in Australia.

The decision on care settings is based on the physical, psychological, medical, cultural, social and restorative care needs of the client. The ACAT also considers the client’s usual accommodation arrangement, financial circumstances, need for assistance, and access to transport and community support systems. The needs of the carer and the client’s personal choice are also taken into account. As part of the initial assessment and needs identification, the ACAP toolkit for assessors provides a set of appropriately validated tools that are nationally consistent.

Under the policy guidelines, the ACATs should have access to a range of skills and expertise to enable a comprehensive assessment. These can include geriatricians, specialists, GPS, nurses, social workers, physiotherapists, occupational therapists and psychologists. The role of the ACATs is to evaluate and recommend appropriate care programs and settings for older Australians. Face-to-face assessment is the core component of an ACAP assessment and can be in the presence of family members or other carers.

This nationally consistent assessment program provides a unique opportunity to analyse the relationship between informal care relationships and recommendations for home or residential care. These data are available at a time in which there is much reform in the Australian aged care system, due to many of the longer term financing and workforce issues also facing long term care in the US [13].

## Methods

### Study population

For this study, we utilise unique unit record de-identified administrative data on aged care assessments from the National Aged Care Data Clearing House, collected between 2010 and 2013 [14]. Before commencing analysis, considerable attention was given to data cleaning and validation. Firstly, as our interest is in movement from the general community into residential care, we limit the population to those assessed in their residence living in the community. Following, we removed records where the assessment was not complete, a duplicate or with missing assessment date data. A small number of records with missing fields on gender and carer measures were also excluded. As an individual may have multiple assessments, the most recent ACAP assessment record for each individual was selected. Removing these duplicate, incomplete and out of scope records, yielded a final sample of approximately 280,000 ACAP assessments for those aged 65 years and over.

### Caring

The definition of carer availability provided in the ACAP data is “whether someone, such as a family member, friend or neighbour, excluding paid or volunteer carers organised by formal services, has been identified as providing regular and sustained care and assistance to the person without payment other than a pension or benefit” [14]. Under the *Carer Recognition Act (2010)*, these caring responsibilities can include assistance with personal care, support and assistance to a person with a disability, medical condition, mental illness or for the frail and aged.

Three categories of caring are available in the ACAP data, including presence of a carer, co-residence of the carer, and relationship of the client to the carer. We utilise these three variables to create a measure of caring in the ACAP process which incorporates carer relationship and co-residence. Specifically, we measure an interaction between carer presence (yes or no), carer relationship status (partner, parent, child, in-law, other relative or friend/neighbour) and co-residence (yes or no).

### Socio-demographic factors

During the ACAP assessment, the assessor collected a range of socio-demographic information on the client. This included cultural identity (Indigenous, born overseas), age and gender of the person, as well as measures of co-residence with other household members. Our categorisation of informal care includes this coresidence component. Including socio-demographic controls is important as Australian and international studies show age, gender, country of birth and coresident living arrangements to be associated with entry to care [6, 7, 15–17].

### Health and assistance needs

A range of health information is collected and coded using the WHO International Classification of Diseases (ICD) framework. We use these variables to measure the client’s number of unique ICD health conditions. Again, numerous studies have pointed to the association between health conditions and entering residential care [6, 16, 18].

Previous studies show measures of Activities of Daily Living (ADL) are among the strongest predictors of entry to care [18, 19]. The measures available in the ACAP data provide important measurement of the functional status of older Australians, with specific reference to their needs for assistance with a range of tasks including body movement, communication, health and other needs (see Table 1).

**Table 1:** ADL Assistance Needs Definition

ADL	ADL Description
	*Needing the assistance or supervision of another person with:*
Body Movement	Activities such as maintaining or changing body position, carrying, moving and manipulation objects, getting in or out of bed or a chair
Moving	Walking and related activities, either away from home or away from home
Communication	Understanding others, making oneself understood by others
Health	Taking medication or administering injections, dressing wounds, using medical machinery, manipulating muscles or limbs, taking care of feet (includes a need for home nursing and allied health care, such as physiotherapy and podiatry)
Self Care	Daily self-care tasks such as eating, showering/bathing, dressing, toileting and managing incontinence
Transport	Using public transport, getting to and from places away from home or driving
Social	Shopping, banking, participating in recreational, cultural or religious activities, attending day centres, managing finances and writing letters
Domestic	Household chores such as washing, ironing, cleaning and formal linen services
Meals	Meals, including the delivery of prepared meals, help with meal preparation and managing basic nutrition
Home	Home maintenance and gardening
Other	Needs assistance with other tasks not stated above

Source: AIHW, 2014

### Outcome variable

Following the ACAP assessment, the outcome of the recommended long term care settings are recorded in the de-identified data file. For this population of older persons living in a private residence, approximately 57% or recommendations are for care in their family home, and a further 41% are for care in residential care. This accounts for over 98% of all recommendations to this group. The final 2% of recommendations are spread amongst independent living, other institutional care and hospitals.

Using this measure of recommended long term care setting, we generated a dichotomous measure. The first group (coded 0) were recommended to obtain care in their private residence. The second group (coded 1) were recommended to obtain care in a residential care setting. This variable is the dependent variable for the logistic regression analyses.

### Statistical analyses

To model the association between carer characteristics and the probability of residential care recommendations, we fit logistic regression models utilizing Stata 14.0. Initial variable selection was informed by the growing literature on the risk factors of entry to residential care, as outlined above. Variables were entered into the regression and improvement to model fit assessed using the Bayesian Information Criteria following Raftery’s procedure [20]. For both male and female samples, separate regressions are estimated, as previous studies have shown differential determinants and size effects by gender [6].

With the gender specific regressions specified, we check the conditioning of the matrix of independent variables to investigate any collinearity influence [21]. The condition numbers were very small providing support for the model specification. Final goodness-of-fit was confirmed using the Hosmer and Lemeshow test [22]. Results by gender were combined into a single parameter vector, to enable testing of regression coefficients between male and female models [23]. Chow tests confirmed that separate male and female models needed to be fitted, as opposed to an aggregate model combining all observations [24] (Chow = 334.98, *p* < 0.001). We also conduct Wald tests of individual coefficients between male and female models to ascertain any gender differences.

## Results

Before turning to the key results, we firstly present descriptive data on patterns of caring collected during ACAP assessments (Table 2). Firstly, it is clear that there are marked gender differences in carer relationships. Indicative of the gender gap in life expectancy, males are more likely to have a partner/wife carer (47%) compared with females (20%). Due to their higher survival, females are in turn more likely to cite carer support from a daughter (35%), or son (18%) when compared to males (18% and 10% respectively). Both groups have similar low proportions of care provision from in-laws, other relatives or friends and neighbours.

**Table 2:** Carer Relationship and Carer Co-residence by Client Gender (%)

	Male (%)	Female (%)	
*Carer Relationship*			
No Carer	14.7	14.7	
Female Partner/Wife	46.9	0.2	***
Male Partner/Husband	0.2	19.7	***
Mother	0.1	0.0	***
Father	0.0	0.0	***
Daughter	17.9	35.4	***
Son	10.2	18.3	***
Daughter In Law	0.8	2.0	***
Son In Law	0.1	0.2	***
Other Relative - Female	3.2	4.4	***
Other Relative - Male	1.7	1.7	
Friend/Neighbour - Female	2.9	2.4	***
Friend/Neighbour - Male	1.2	0.9	***
Total	100	100	
*Carer Co-residence*			
No Carer	14.7	14.7	
Carer Co-resident	55.7	35.4	***
Carer Non Co-resident	29.6	49.9	***
Total	100	100	
*n*=	114,894	166,549	

Notes: *** *p* < 0.001for test of proportion between male and female samples

Across all relationship categories, females are more likely to care than males. For example, for male clients about 18% receive care from a daughter relative to 10% from a son. The gender differences are considerably higher for female clients (35% receive care from a daughter compared with 18% receiving care from a son). Again reflective of differential survival prospects, men are more likely to have a co-resident carer (56%) when compared to women (35%). Overall, equal proportions of males and females accessed through the ACAP program have no carer (about 15%).

Using these measures (Table 2), we generated a variable which measures the interaction between carer relationship (no carer, partner, parent, child, child in-law, other relative or friend/neighbour) and co-residence. Table 3 present the ACAP recommendation (private residence versus residential care), tabulated by carer relationship and co-residence along by gender. Also included are a range of background demographic, health and functional needs measures.

**Table 3:** Carer, Demographic and Health Characteristics by Gender and ACAP Recommendation (%)

	Males (%)			Females (%)		
	Private	Residential		Private	Residential	
*Carer Residence & Relationship*						
No carer	14.1	15.3	***	16.0	12.6	***
Partner - cores	50.9	41.1	***	22.0	16.4	***
Partner - non cores	0.6	0.7		0.3	0.2	
Parent - cores	0.1	0.1		0.0	0.0	
Parent - non cores	0.0	0.0		0.0	0.0	
Child - cores	6.7	6.8		13.4	13.3	
Child - non cores	19.2	24.0	***	38.2	43.6	***
Child In Law - cores	0.3	0.3		0.4	0.5	
Child in Law - non cores	0.6	0.8	***	1.6	1.9	***
Other relative - cores	0.9	1.2	***	1.2	1.4	***
Other relative - non cores	2.9	5.1	***	3.9	6.2	***
Friend - cores	1.0	1.0		0.6	0.6	
Friend - non cores	2.6	3.7	***	2.4	3.1	***
*Cultural Identity*						
Australian Born	62.9	66.1	***	66.5	71.3	***
Indigenous	0.9	0.7	**	0.9	0.8	***
Born Overseas	36.2	33.1	***	32.6	27.9	***
*Age*						
65–74	18.7	15.6	***	16.1	10.5	***
75–84	46.5	42.1	***	44.7	37.8	***
85+	34.8	42.3	***	39.2	51.7	***
*Assistance Needs*						
Self	65.2	88.3	***	61.6	86.1	***
Move	20.7	46.8	***	18.4	40.1	***
Moving	53.4	75.1	***	54.7	73.9	***
Communication	15.9	29.8	***	11.4	24.4	***
Health	78.5	92.9	***	73.5	90.3	***
Transport	84.0	95.3	***	90.2	97.1	***
Social	82.1	92.8	***	84.9	94.1	***
Domestic	96.1	95.3	***	95.0	95.5	***
Meals	88.1	93.6	***	78.2	91.8	***
Home	82.0	80.6	***	82.5	81.1	***
Other	5.4	6.0	***	5.7	6.6	***
*Number of ICD Conditions*						
0	0.2	0.1	***	0.2	0.0	***
1–3	20.6	16.7	***	19.7	15.7	***
4–6	62.7	65.4	***	63.6	65.8	***
7+	16.6	17.8	***	16.6	18.4	***

Notes: cores – coresidence. *** *p* < 0.001 ** *p* < 0.01 * *p* < 0.05; cores – co-resident; *ICD* International Classification of Diseases

Compared to males with a private residence recommendation, those with a recommendation for residential care were less likely to have a co-resident partner (51% v 41%) and more likely to rely on care from a non-coresident child (24% v 19%), other non-coresident relative or a non-coresident friend. For females, this relationship is broadly replicated (a higher proportion of non-coresident care for those with a residential recommendation), although the size of differences is remarkably different. For example, 16% of females with a residential recommendation have a partner coresident carer compared with 22% of those in a private residence. Moreover, when a child is providing care, they are more likely to provide non-coresident care, for both men and women, although the gap again for women is larger. For example, for women with a residential care recommendation, 44% of women have a non-coresident child carer and 13% have a coresident child carer – yielding a coresidence gap of 30%. The gap for men with a residential care recommendation is about half this amount, 17%.

Among the socio-demographic control variables, those with a recommendation for residential care are more likely to be Australian born and non-indigenous, to be older, to have a higher number of health conditions and also, considerably more likely to have a number of functional needs limitations. These differences are consistent by gender, however the size of differences, once again, vary.

To disentangle the impact of co-residence, carer relationships and control variables (demographic, health and functional needs) on assessor recommendations, we estimated logistic regression models with the assessor recommendation (private residence versus residential care) as the dependent variable (Table 4). The analyses are separated by gender (male, female) and the column titled ‘Test Gender’ is the linear Wald test of differences in coefficients between male and female models. That is, a test of whether a particular caring relationship has a stronger effect on the assessor’s recommendations for men relative to women.

**Table 4:** Logistic Regression Model of Assessor Recommendations

	Male				Female				Test Gender
	OR	*p*-value	[95% CI]		OR	*p*-value	[95% CI]		
*Carer Residence & Relationship*									
No carer	1.00				1.00				
Partner - cores	0.37	0.000	0.36	0.39	0.58	0.000	0.56	0.60	***
Partner - non cores	0.73	0.000	0.62	0.86	0.85	0.140	0.68	1.05	
Parent - cores	0.62	0.048	0.38	0.99	0.45	0.005	0.25	0.79	
Parent - non cores	1.26	0.680	0.42	3.79	0.80	0.602	0.35	1.82	
Child - cores	0.47	0.000	0.45	0.50	0.68	0.000	0.65	0.71	***
Child - non cores	0.84	0.000	0.80	0.87	1.06	0.001	1.02	1.09	***
Child In Law - cores	0.50	0.000	0.39	0.64	0.76	0.001	0.65	0.89	**
Child in Law - non cores	0.84	0.028	0.72	0.98	1.02	0.603	0.94	1.11	*
Other relative - cores	0.69	0.000	0.61	0.78	0.87	0.005	0.79	0.96	**
Other relative - non cores	1.15	0.000	1.07	1.23	1.47	0.000	1.40	1.55	***
Friend - cores	0.54	0.000	0.48	0.62	0.78	0.000	0.68	0.89	***
Friend - non cores	0.91	0.029	0.84	0.99	1.17	0.000	1.09	1.26	***
*Cultural Identity*									
Australian Born	1.00				1.00				
Indigenous	0.85	0.024	0.73	0.98	0.90	0.084	0.80	1.01	
Born Overseas	0.85	0.000	0.83	0.87	0.79	0.000	0.77	0.81	***
*Age*									
65–74	1.00				1.00				
75–84	1.18	0.000	1.14	1.23	1.29	0.000	1.25	1.34	***
85+	1.48	0.000	1.43	1.54	1.73	0.000	1.67	1.79	***
*Assistance Needs*									
	1.60	0.000	1.58	1.61	1.58	0.000	1.57	1.59	*
^a^Number of ICD	0.98	0.014	0.97	0.99	1.01	0.028	1.00	1.02	**
ICD^a^2	0.99	0.000	0.98	0.99	0.99	0.000	0.99	0.99	
ICD^a^3	1.00	0.014	1.00	1.00	0.99	0.058	1	1.00	**
Observations	113,379				164,167				
Log Link	17,219.26				22,284.12				
Chow Test (20df)	334.98	***							

Notes: *** *p* < 0.001 ** *p* < 0.01 * p < 0.05; cores – co-resident; *ICD* International Classification of Diseases, *OR* adjusted odds ratio, *95% CI* 95% confidence interval for the odds ratio; Test Gender – wald tests of male coefficient versus female coefficient. ^a^ Number of ICD conditions entered with nonlinear terms

For both males and females, living with a coresident partner is strongly associated with a recommendation to remain living in the community. Males with a coresident partner are about 63% less likely and females about 42% less likely to have a residential care recommendation, compared with those with no carer. The effect of a coresident partner appears to be more protective for men than women (*p* < 0.001), although the effect for women remains very strong.

The presence of a coresident child carer is also strongly associated with a community versus residential recommendation is. Males with this form of care are 53% less likely, and females 32% less likely to have a residential care recommendation. Again, the impact of a child coresident carer appears stronger for males than females (*p* < 0.001). Interestingly, for males, the impact of having a coresident child-in law is the same (OR = 0.50 *p* < 0.01). The effect for females is again significant, but weaker (OR = 0.76 p < 0.01). Care provided by a non-coresident parent did not alter the likelihood of assessor recommendation for males or females (*p* > 0.1). For females, presence of a non-coresident child in law is also not associated with assessor recommendations.

Despite these similarities in the effects of carer relationships by gender, there are some notable exceptions. For females, receipt of care from a non-coresident other relative is associated with a residential care recommendation (OR = 1.47 *p* < 0.001). That is a relative other than a partner, in-law or child. Women with this form of care are about 47% more likely to have a residential recommendation rather than a community recommendation. This effect is significant for males, but weaker (Wald *p* < 0.01). For females also, having a non-coresident carer who is a friend or neighbour is more likely to have a residential care recommendation (OR = 1.17 *p* < 0.001), whereas for males the effect is in the opposite direction and weak (OR = 0.91 *p* < 0.05).

Independent, of these caring effects, cultural identity, age, assistance needs and health conditions are all strongly associated with assessors recommendations for care settings. Consistent with other studies, increasing assistance needs and health conditions are associated with assessor recommendations. For the number of ICD conditions, nonlinear terms are added to the model which show that the probability of having a residential care recommendation maximises at around 5 to 6 ICD condition types, and slightly tapers thereafter. These nonlinear terms are centered to remove any multicollinearity influence. More detailed modelling (omitted here) show that assistance needs types are among the strongest influences on assessors’ care recommendations.

## Discussion

Previous international and Australian studies have provided mixed findings on the role of informal care (and care proxies) on entry to residential care [6–11]. Some studies have found informal care provision to increase the likelihood of entry to residential care, while others find the opposite and some studies find no impact at all. Although these studies differed in country and policy settings, data collection methodologies and measurement, the differences in findings appeared puzzling given the accepted opinion in Australia that informal care protects against entry to residential care, despite a lack of statistical modelling. Providing part of the explanation for these divergent findings in the literature, we use a detailed measure to show that some carer relationships and residence are associated with recommendations to live in the community, some to live in residential care, and some care types have no association with ACAT assessor recommendation at all.

Specifically, the impacts of coresident caring (regardless of relationship) is important for a private residence recommendation, but has the strongest effect for males. For both males and females, coresident child and partner caring play the strongest protective effect on a residential care recommendation. However, not all caring relationships are protective of a residential care recommendation. For both males and females, non-coresident other relatives are associated with a residential care recommendation. Similarly, for females, receipt of non-coresident care from a friend or neighbour is also associated with a residential care recommendation. Finally, some care relationships, such as non-coresident child in-law (for females) or non-coresident parent care, have no impact on the assessor recommendations, although these caring relationships are rare.

This raises the question of why assessor recommendations diverge based on informal care relationships? It is clear that ACAT assessors (either subjectively or objectively) determine that close familial caring relationships, particularly in the household, is what is important for keeping people independent in the community. Looser relationships, such as friendship of neighbour care are perhaps seen as more temporary in nature, without filial obligations to continue a caring role. Care provided by non-coresident intermediate relatives (children in-laws) is seen by assessors as not key to their recommendations. Although speculative, part of the explanation may be differences in care frequency, the carer’s age and carers care obligations to their immediate family. More generally, with a co-resident carer, assessors are less likely to be concerned about the risks of falls and having no-one to immediately assist.

The strength of coefficients between the models also show that co-resident partner and child care provision is more important for males than females in the assessors’ recommendations. Again, although speculative, it may be that in this cohort of older persons, this generation of males have lower skill sets in basic household and personal care tasks relative to women. It may also be that partner care in particular, is considered the normative care mode for males, but not for females.

Overall, although the impacts of carer relationships on assessors’ recommendations are strong and highly significant, the effects of assistance needs (particularly self care, move and health) and multiple ICD health conditions play a very strong role in the assessors’ recommendations to residential care. This is strongly consistent with both Australian and international studies [18, 19]. Age has a stronger impact on females’ propensity to be recommended to residential care, relative to males.

Apart from providing an explanation of the differential effects of carer relationships and coresidence on residential care recommendations, results from this study are important for other reasons. In Australia, as in many other industrialised nations, there are several demographic trends that have implications for ongoing care provision, specifically given the importance of carer relationships observed in this study. Firstly, survival prospects in Australia show considerable improvement over the last 30 years, leading to longer lives for men in particular [25]. Related to this change, Australian studies are projecting a significant increase in the number of men living alone [26]. Furthermore, given the long term trend of fertility decline in Australia, average number of children ever born by future cohorts of older persons will be lower [27, 28]. Moreover, relative to the past, many adult children are living further distances from their parents, with implications for the provision of care [29]. Approximately one third of caregivers in Australia are aged over 60 and the average age of primary caregivers in Australia is 55 years [30]. There is also a trend for increased workforce participation amongst women in this age group [31]. These demographic trends, taken together suggest that the provision and distribution of carer relationships in future cohorts of older persons may be very different to what is observed today. This has clear implications for the propensity for residential care admission in the future, particularly given that it is coresident partners and coresident children that most strongly protect against recommendations to enter residential care.

One limitation of this study is that although the ACAT collects information on the level of carer stress, this is not provided to researchers in the de-identified administrative data. This is important as US studies show that carer stress is a strong predictor of nursing home entry [32]. Indeed, Australian studies show that many informal carers are in a position of stress due to the indirect and direct costs incurred in their caring role [33]. A further limitation is that relative to other studies that measure entry to residential care, we measure the first compulsory formal in gaining access to residential care in Australia, the ACAP assessment. Following the ACAP assessors recommendation and subsequent approval to enter residential care, the individual applies to enter a residential care facility. Future studies should examine the role of carer relationships and co-residence at the point of admission.

In recent years, the aged care system in Australia has undergone considerable reform, with the introduction of a consumer directed care (CDC) model, a model of service delivery designed to give more choice and flexibility to consumers and a further shift in the balance of government spending to home rather than residential care [34]. Currently the CDC model applies to recipients of home care packages, which are provided at four levels depending on the assessed needs of the older person but in future it will apply to residential care as well. The data we use pre-date the CDC model. With future releases of the ACAP administration data, a priority is to examine whether assessors recommendations have changed.

## Conclusions

Noting these limitations and extensions, results from this study are important as they provide some evidence as to why some studies show informal care to be positively associated with entry to residential care, others negatively and some studies showing no effect at all. Carer relationships and coresidence alter the pathways to care as recommended by assessors in Australia. Consistent with the work of others, these results suggest that more detailed measures of informal care (such as carer stress, family assistance and the quality of care) would help better understand the complex relationship between informal and formal care provision [12].

Moreover, results herein are important for government planning and policies. Consistent with its long-standing recognition of the important role of caregivers in caring for people with disability and frail older people, the Australian government provides a range of support services for family caregivers, including means tested pensions, non-means tested payments, respite care, carer support help lines and advocacy services. Given the long-standing policy preference for aged care to be delivered in the home rather than residential care settings, the need to support caregivers should continue to be a government priority. However, our findings suggest that policy consideration should be given to the differential effects of coresident versus non-coresident caregivers in enabling older men and women to remain in their own homes. In the light of our findings, the Australian government should consider how coresident carers can be given additional support. This could be in the form of rental relief; in-home respite care to enable them to participate in the workforce; and/or support to manage the older person’s personal care needs. With future releases of the ACAP administration data, a priority is to examine whether assessor recommendations have changed with the move to a CDC model in Australia, particularly as they pertain to informal care provision.

## Abbreviations

ACAP: Aged care assessment program
ACAT: Aged care assessment team
ADL: Activities of daily living
CDC: Consumer directed care
ICD: International classification of diseases
